# Alcohols inhibit translation to regulate morphogenesis in *C*. *albicans*

**DOI:** 10.1016/j.fgb.2015.03.008

**Published:** 2015-04

**Authors:** Nkechi E. Egbe, Caroline M. Paget, Hui Wang, Mark P. Ashe

**Affiliations:** Faculty of Life Sciences, University of Manchester, Manchester M13 9PT, United Kingdom

**Keywords:** *Candida albicans*, Protein synthesis, Filamentous growth, Eukaryotic initiation factor 2B (eIF2B)

## Abstract

Many molecules are secreted into the growth media by microorganisms to modulate the metabolic and physiological processes of the organism. For instance, alcohols like butanol, ethanol and isoamyl alcohol are produced by the human pathogenic fungus, *Candida albicans* and induce morphological differentiation. Here we show that these same alcohols cause a rapid inhibition of protein synthesis. More specifically, the alcohols target translation initiation, a complex stage of the gene expression process. Using molecular techniques, we have identified the likely translational target of these alcohols in *C*. *albicans* as the eukaryotic translation initiation factor 2B (eIF2B). eIF2B is the guanine nucleotide exchange factor for eIF2, which supports the exchange reaction where eIF2.GDP is converted to eIF2.GTP. Even minimal regulation at this step will lead to alterations in the levels of specific proteins that may allow the exigencies of the fungus to be realised. Indeed, similar to the effects of alcohols, a minimal inhibition of protein synthesis with cycloheximide also causes an induction of filamentous growth. These results suggest a molecular basis for the effect of various alcohols on morphological differentiation in *C*. *albicans*.

## Highlights

• Alcohols induce morphological alterations in *C. albicans*.
• Alcohols inhibit protein synthesis.
• Translational inhibition occurs as a result of eIF2B regulation.
• Regulation of protein synthesis and morphogenesis are mechanistically connected.

## 1 Introduction

*Candida albicans* is a major fungal pathogen of humans, causing life threatening septicaemic infections, especially in immunocompromised individuals. In addition, *C*. *albicans* can cause frequent and recurrent infections that are difficult to treat in healthy individuals. *C*. *albicans* has a variety of properties that have been implicated in pathogenicity, including the ability to switch growth between a variety of morphological forms, such as yeast, pseudohyphal and hyphal forms. Such switches in morphology occur as a response to environmental cues, where external stimuli are communicated via different signal transduction pathways (Biswas et al., 2007; Brown and Gow, 1999; Liu, 2001). Pseudohyphae are morphologically distinguishable from hyphae; they also differ fundamentally in their cell cycle organisation and in mechanisms of polarised growth (reviewed in Sudbery (2011)).

A variety of yeasts secrete alcohols such as butanol and isoamyl alcohol during growth (Dickinson et al., 1998, 2003; Ghosh et al., 2008; Hazelwood et al., 2008; Martins et al., 2007; Webb and Ingraham, 1963). These alcohols are major components of fusel oil; a by-product of yeast fermentation, and hence they have been collectively termed fusel alcohols (Webb and Ingraham, 1963). The addition of fusel alcohols to a yeast culture has been shown to induce a range of specific morphological effects such as pseudohyphal growth. It has been suggested that these alcohols might signal nitrogen scarcity to elicit these effects (Dickinson, 1996). Consistent with this hypothesis, both non-pathogenic and pathogenic yeasts switch from the budding yeast form to the filamentous form when starved for nitrogen (Csank and Haynes, 2000; Gimeno et al., 1992).

The role of fusel alcohols and other alcohols in signalling to alter the growth and physiology of *Candida* species is starting to become more widely appreciated. Martins et al. (2007) detected Isoamyl alcohol, 2-phenethylethanol, 1-dodecanol in supernatants of planktonic and biofilm forms of *C*. *albicans* and *C*. *dubliniensis*, where these alcohols inhibited the morphological transition from yeast to filamentous form by over 50%. Aromatic alcohols i.e. phenethyl alcohol, tyrosol and tryptophol are produced by *C*. *albicans*, especially under nitrogen poor conditions (Chen and Fink, 2006; Chen et al., 2004; Ghosh et al., 2008; Lingappa et al., 1969; Martins et al., 2007). These aromatic amino alcohols induce pseudohyphal formation in *Saccharomyces cerevisiae*, with tyrosol also inducing germ-tube formation in *C*. *albicans* (Chen and Fink, 2006).

In *S*. *cerevisiae* a number of examples exist where nutritional alterations induce filamentous growth and also affect protein synthesis. Hence, nitrogen limitation affects translation initiation and also induces pseudohyphal growth in diploids (Gancedo, 2001; Gimeno et al., 1992; Ibrahimo et al., 2006). Moreover, the effects of various alcohols as well as glucose depletion have been shown to modulate both protein synthesis and filamentous growth in *S*. *cerevisiae* (Ashe et al., 2000, 2001; Lorenz et al., 2000).

Translation initiation is the predominant phase where protein synthesis is regulated. The process of translation initiation requires interaction of the initiator methionyl tRNA with eukaryotic initiation factor 2 (eIF2) bound to GTP to form a ternary complex. This is then recruited to the small ribosomal subunit (40S) to form the 43S complex. Following the binding of the 43S complex to the mRNA and start codon recognition, the GTP on eIF2 is hydrolysed and released as eIF2-GDP (Hershey and Merrick, 2000). The initial formation of the ternary complex represents a prominent point of translational regulation across all eukaryotes. For instance, in *S*. *cerevisiae* activation of the eIF2α kinase, Gcn2p, during amino acid starvation leads to the phosphorylation of the α subunit of eIF2. This phosphorylated form binds tightly and sequesters eIF2B; the guanine nucleotide exchange factor that is responsible for recycling eIF2-GDP to eIF2-GTP. Hence, the inhibition of eIF2B results in less eIF2-GTP, less ternary complex and reduced levels of global protein synthesis (Hinnebusch, 2005; Pavitt, 2005). Even though regulation of eIF2B results in a global regulation of translation, specific mRNAs such as *GCN4* are activated in a mechanism requiring four upstream open reading frames (uORFs) (Hinnebusch, 2005). *GCN4* encodes a transcription factor that regulates the expression of a host of genes involved in the regulation of nitrogen resources in cells (Natarajan et al., 2001). Therefore Gcn4p up-regulation reverses the starvation by promoting amino acid biosynthesis as part of the general control pathway. *C. albicans* also harbours a single eIF2α kinase, Gcn2p (Tournu et al., 2005). As in *S*. *cerevisiae*, amino acid starvation inhibits global translation via this kinase while activating translation of *GCN4*, yet in this case there are just three uORFs involved in the regulation (Sundaram and Grant, 2014b).

In this current study, we have assessed the translational and morphological responses of *C*. *albicans* to the effects of various alcohols. We confirm previous analyses showing that fusel alcohols and ethanol induce a switch from vegetative growth to pseudohyphal growth. We show that the alcohols also rapidly inhibit translation initiation and induce the translation of the *GCN4* mRNA in both the wild type and the *gcn2Δ* strains. Therefore, Gcn2p is not involved in this pathway of translation regulation in *C*. *albicans* and a pathway that is independent of the eIF2α kinases must regulate translation to induce *GCN4* expression. Given the known effects of fusel alcohols targeting eIF2B in *S*. *cerevisiae*, these results are entirely consistent with the presence of a similar mechanism in *C*. *albicans* and opens up the possibility that such a regulation could be involved in morphogenetic alterations and pathogenicity. Indeed we show that minimal inhibition of translation with cycloheximide leads to a similar degree of filamentation as alcohol treatment.

## 2 Materials and methods

### 2.1 Media and growth conditions

The *Candida* strains used in this study are listed in Table 1. The CAI4 background is used throughout (Fonzi and Irwin, 1993), however experiments carried out comparing the CAI4 and CAF2-1 strains showed that the two strains responded similarly to various alcohol treatments in terms of growth and filament formation (data not shown). Therefore, as CAI4 is the parental strain for the *gcn2Δ* mutant, this strain was selected for use in this study. The strains were grown and maintained on rich yeast extract-Peptone-Dextrose (YPD) media (2% (w/v), glucose; 2% (w/v) bactopeptone; and 1% (w/v), yeast extract) or YPD agar solid media (YPD with 2% agar). Unless otherwise stated, alcohols (butanol, isoamyl alcohol and ethanol) were added for 15 min at the particular concentrations stated. Alcohol tolerance was assessed by growing strains in YPD to OD_600_ 0.1. Butanol (0.5%, 1% and 2%), isoamyl alcohol (0.1%, 0.25%, 0.5% and 1%) and ethanol (2%, 4%, 6% and 8%) were added and growth was assessed hourly.

### 2.2 Morphogenesis assays

Strains were grown at 30 °C, washed in water to remove trace media constituents and re-inoculated into fresh YPD medium supplemented with alcohols to OD_600_ 0.1 then incubated at 37 °C. As positive and negative controls, cells were re-inoculated into 10% serum medium or YPD lacking alcohol, respectively. At various times during incubation (1 h, 2 h, 3 h, 4 h), the morphology of the cells was monitored using a Nikon Eclipse E600 and Axiocam MRm camera. Images were acquired using Axiovision 4.5 software. The proportion of cells forming germ tubes or pseudohyphae were counted using a haemacytometer. Each count was repeated three times and the mean of the three counts recorded. Colony morphology on solid media was assessed by plating on YPD agar or YPD agar supplemented with the indicated alcohol, 10% serum or cycloheximide concentrations ranging from 10 μg/ml to 200 μg/ml. Micro-colonies were photographed using a Nikon Eclipse E600 and Axiocam MRm camera and the images were acquired using Axiovision 4.5 software.

### 2.3 Polysome analysis

*C*. *albicans* strains were grown to an OD_600_ of 0.7 and treated with butanol, isoamyl alcohol and ethanol as described above. Extracts were prepared in 1 mg/ml cycloheximide and these were layered onto 15–50% sucrose gradients. The gradients were sedimented via centrifugation at 40,000 rpm using a SW41 Beckman rotor for 2.5 h (Fullerton, CA) and the *A*_254_ was measured continuously to give the traces shown in the figures (Ashe et al., 2000).

### 2.4 [^35^S] methionine incorporation assay

*C*. *albicans* strains were grown to OD_600_ of 0.7 in synthetic complete dextrose (SCD) medium lacking methionine (Guthrie and Fink, 1991). The culture was split into two flasks and methionine was added to a final concentration of 60 ng/ml, of which 0.5 ng/ml was [^35^S] methionine (cell-labelling grade 1175 Ci/mmol; New England Nuclear, Boston, MA). Alcohol at the indicated concentration was added to one flask and samples (1 ml) were taken and processed as described previously (Ashe et al., 2000). For each experiment, three biological replicates were assessed.

### 2.5 Western blot analysis of phosphoserine 51 eIF2α

50 ml OD_600_ 0.7 of culture grown in YPD was harvested in a clinical centrifuge at 5000 rpm for 5 min. The cells were lysed, and protein samples were prepared as described previously (Ashe et al., 2000; Taylor et al., 2010). Immunoblots were probed using phosphospecific eIF2α antibodies and antibodies raised against yeast elongation factor 1 (Tef1p) as a loading control.

### 2.6 Reporter assays

Luciferase assay was performed essentially as explained previously (Srikantha et al., 1996). Briefly, cells were harvested and extracted using RLUC buffer (0.5 M NaCl, 0.1 M K_2_HPO_4_ (pH), 1 mM Na2EDTA, 0.6 mM sodium azide, 1 mM phenylmethylsulfonyl fluoride, 0.02% bovine serum albumin). The assay was initiated by addition of 1.25 μM coelentrazine h (Promega) to cell extracts, and activity was measured using GloMax 20/20 luminometer (Promega). Luciferase activity (RLU) is expressed as relative luminescence per 10 s/mg protein. As a control, Luciferase mRNA was evaluated relative to GAPDH mRNA by qRT-PCR and no significant changes were elicited by any of the treatments used (data not shown).

### 2.7 Microscopy

Real-time 2D deconvolved projections from continuous z-sweep acquisition were generated using a Delta Vision RT microscope (Applied Precision, Isaaquah, WA) with an Olympus 100× 1.40 NA DIC oil PlanApo objective (Melville, NY) and Roper CoolSnap HQ camera (Tucson, AZ) using Applied Precision Softworx 1.1 software and 2 × 2 binning at room temperature. Z-sweep acquisition allowed fast visualisation of all planes while minimising fluorescent bleaching. Time-course experiments were performed by acquiring images every 5 s over 2 min. ImageJ (http://rsb.info.nih.gov/ij/; NIH) was used to track the movement of the 2B body on the images acquired over the time course. The values calculated for the mean total distance moved by the 2B body were subjected to statistical analysis via a two-sample *t* test assessing significance after alcohol treatment relative to untreated cells.

### 2.8 Alcohol analysis

Strains were grown in YPD media in both aerobic flasks and semi-anaerobic vials until steady state was reached. 2 ml samples were passed through a 0.22μ filter into gas-chromatography (GC) vials and analysed by GC-FID using an Agilent 6850A GC system with an automatic injector, sampler and controller (Agilent 4513A). A DB-WAX capillary column (30 m × 0.25 mm, 0.25 μM, J & W Scientific) was used for separation. Samples were quantified relative to a standard of ethanol, isoamyl alcohol and isobutanol and butanol.

## 3 Results

### 3.1 Growth and protein synthesis are inhibited in response to alcohols

The response of *S. cerevisiae* and *C*. *albicans* to alcohols varies greatly: alcohols can induce morphological changes including pseudohyphal and hyphal growth (Ashe et al., 2001; Chen and Fink, 2006; Dickinson, 1996; Ghosh et al., 2008), or the alcohols can prove toxic leading to cell death (Kitagaki et al., 2007). Alcohols, under certain culture conditions, have also been reported to inhibit filament formation in various *Candida* species (Chauhan et al., 2011, 2013; Martins et al., 2007). As a result it has been suggested that these alcohols are produced by yeast species to act as concentration dependent signals. We investigated the level of such alcohols in the CAI-4 strain of *C*. *albicans* grown on rich media (YPD) and found that over a period of 2 days aerobic growth, levels of ethanol, isoamyl alcohol and isobutanol accumulated to 0.74% (5.88 g/l), 111 ppm (90 mg/l) and 34 ppm (27 mg/l) respectively. Under anaerobic conditions, over a period of 6 days, the level of ethanol and isobutanol accumulated to 1.2% (9.5 g/l) and 22 ppm (18 mg/l) respectively, whereas isoamyl alcohol was only detected later after 14 days at 87 ppm (70 mg/l).

**Fig. 1:**
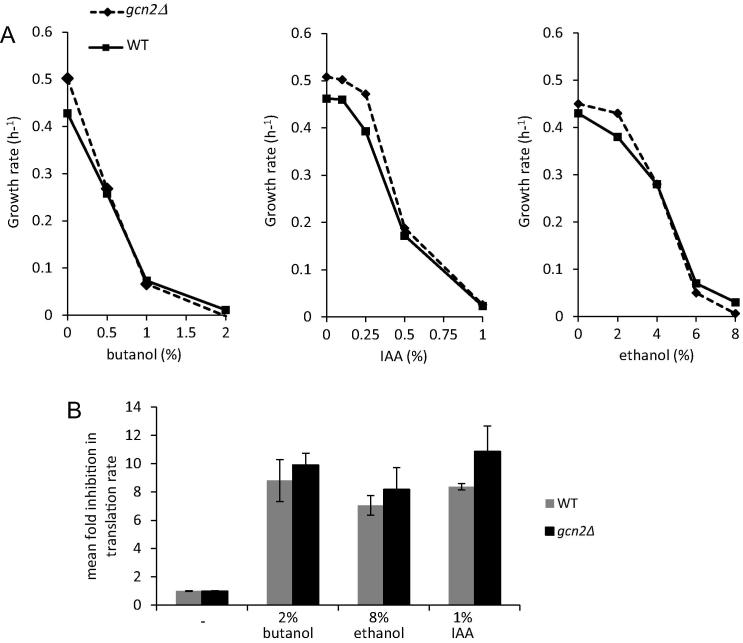
Concentration dependent inhibition of growth and protein synthesis by alcohols does not rely on *GCN2*. (A) Figure shows plots of growth rates at various concentrations of ethanol, butanol and isoamyl alcohol (IAA) as indicated. Wild type CAI4 strain and the mutant *gcn2Δ* strain were grown to OD_600_ of 0.1, then treated and the OD_600_ was taken hourly for 6 h to generate the growth rates plotted. (B) A bar chart depicting the fold inhibition in the rate of protein synthesis at the indicated concentrations of alcohol for wild-type and *gcn2Δ* cells. Translation rates were determined by measuring using ^35^S methionine incorporation over a 10 min period. Each treatment was analysed in triplicate; error bars are ±SEM.

In order to analyse the possible implications of alcohol accumulation on the growth and morphology of the CAI-4 strain of *C*. *albicans*, the response to added *n*-butanol, isoamyl alcohol (IAA) or ethanol was studied. Firstly, in response to *n*-butanol, growth was weakly inhibited by concentrations of 0.5% (v/v), while at 2% (v/v) the growth of the strain was completely inhibited (Fig. 1A). A similar scenario was evident after the addition of IAA and ethanol, only the concentrations causing weak and complete inhibition varied. For instance, 1% (v/v) IAA caused complete growth inhibition (Fig. 1A), whereas higher concentrations of ethanol (6–8% (v/v)) were required to bring about the same effect (Fig. 1A). Therefore, in terms of toxicity, the alcohols prevent growth at concentrations of 2% (v/v) *n*-butanol, 1% (v/v) isoamyl alcohol and 6–8% (v/v) ethanol (Fig. 1A); levels that are significantly higher than can be observed to accumulate in *C*. *albicans*.

The inhibition of growth of *C*. *albicans* by the alcohols used in this study, prompted us to examine the rate of protein synthesis following alcohol treatment. Cells were labelled with [^35^S] methionine for 5 min prior to addition of a particular alcohol, after which incorporation was measured for 10 min. In these experiments, the addition of alcohols (2% (v/v) butanol, 1% (v/v) IAA or 8% (v/v) ethanol) caused a 10-fold decrease in the rate of protein synthesis (Fig. 1B). Therefore, these alcohols inhibit both growth and protein synthesis in *C*. *albicans*.

### 3.2 Alcohols induce filamentation

**Fig. 2:**
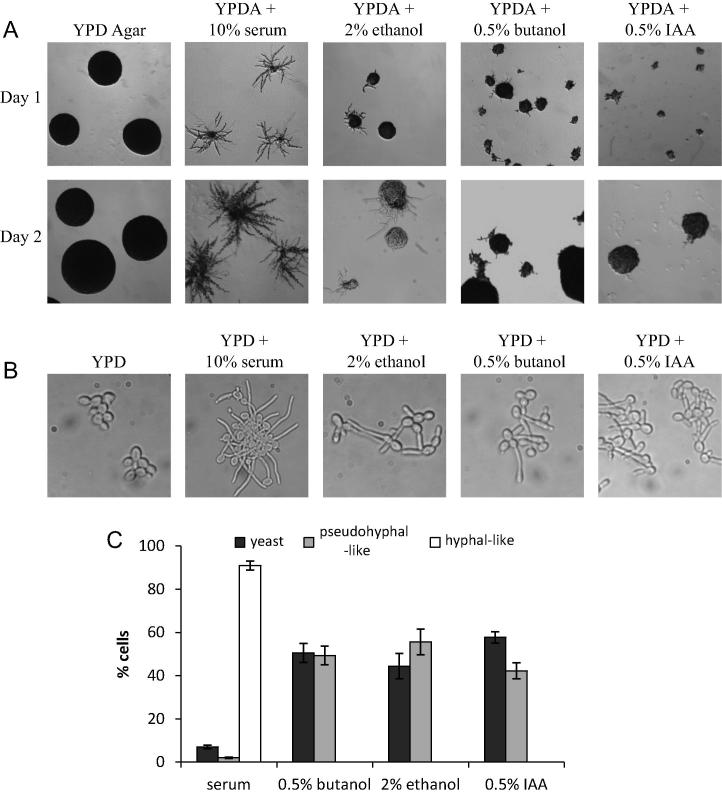
Alcohols cause morphological differentiation in *C*. *albicans*. (A) Serial dilutions of overnight cultures of the wild type CAI4 strain were spread onto YPD agar plates containing 0.5% butanol, 2% ethanol, 0.5% IAA or 10% serum. Representative microcolonies were photographed (10× magnification) after 1 and 2 days of growth as indicated. (B) Images of cells from cultures that were grown at 37 °C in the indicated media for 3 h. (C) Cells from the analysis in B were scored for yeast, hyphal and pseudohyphal growth in a cell counting chamber. Percentages given are an average (±SEM) calculated from three biological replicates.

On the basis of the growth considerations detailed above, an intermediate concentration of each alcohol (0.5% (v/v) *n*-butanol, 0.5% (v/v) isoamyl alcohol or 2% ethanol (v/v) was used to explore the switch from vegetative to filamentous growth. It is well-established that treatment of *C*. *albicans* with serum induces a robust switch to filamentous growth both in solid and liquid media (Feng et al., 1999; Liu, 2001). Indeed, microscopic examination of cells early after serum treatment, suggest that the majority of cells were undergoing filamentous growth (Fig. 2C). All of the alcohols tested cause a switch to filamentous growth at some level (Fig. 2A and B), but the response was significantly less robust than for serum addition. For instance, roughly half the cells switch to a more pseudohyphal-like growth form early after alcohol addition (Fig. 2C), as judged by criteria such as relative cell shape, length and the presence of constriction between cells (Sudbery et al., 2004; Sudbery, 2011). In addition, much less florid micro-colonies are observed on alcohol containing relative to serum containing solid agar (Fig. 2A). Overall these data show that the addition of mildly inhibitory concentrations of *n*-butanol, isoamyl alcohol or ethanol to the growth media of *C*. *albicans* slows the growth rate and induces morphological differentiation both in solid and liquid growth media.

### 3.3 Concentration dependent inhibition of translation initiation

**Fig. 3:**
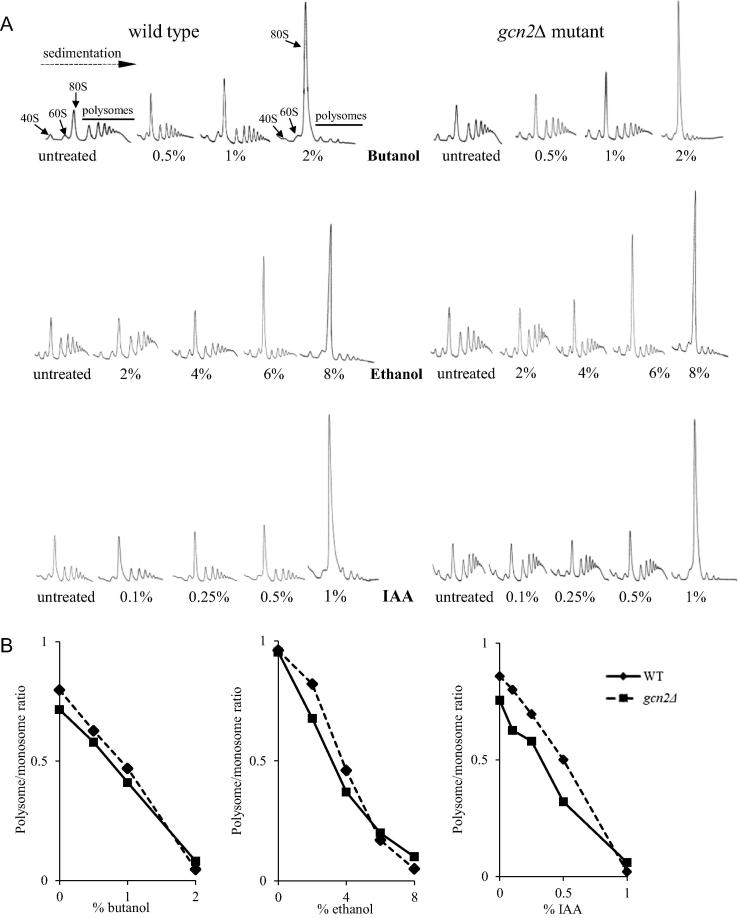
Translation initiation is inhibited by alcohols in CAI4 and *gcn2Δ* strains. (A) Figure shows polysome analyses assessing the effect of alcohols on translation initiation in the CAI4 wild type and *gcn2Δ* strains of *C*. *albicans*. Strains were grown in YPD and the indicated concentrations of the alcohols were added for 15 min prior to extract preparation. Extracts were sedimented on 15–50% sucrose gradients and the absorbance at 254 nm was continuously measured to generate the traces depicted. The position of 40S, 60S, 80S and polysome peaks are labelled and the direction of sedimentation is also depicted. (B) Quantitation of the polysome: monosome ratio across the polysome profiles. Solid lines represent the wild type, dashed lines represent *gcn2Δ* strain.

To further investigate the stage of protein synthesis targeted by the alcohols in *C*. *albicans*, and given that fusel alcohols bring about a rapid inhibition of translation at the initiation step in *S*. *cerevisiae* (Ashe et al., 2001), we set out to investigate if butanol and ethanol exert a similar effect on translation initiation in *C*. *albicans*. Polysome analysis can be used to investigate both the level of protein synthesis occurring in cells and, under stress conditions, what step of the translation pathway is inhibited (Ashe et al., 2001). An analysis of the distribution of polysomes across a sucrose gradient revealed that at increasing concentration of the alcohols, a change in the polysome profiles was observed. The 80S peak increased dramatically and the polysome peaks were reduced (Fig. 3A). This change in profile is termed polysome run-off and is characteristic of an inhibition of initiation step of translation (Ashe et al., 2001; Hartwell and McLaughlin, 1969). Once again, the concentration required to induce these effects varied across the alcohols tested (Fig. 3B). Butanol and IAA effectively inhibit translation at 2% (v/v) and 1% (v/v) respectively, whereas much higher concentrations of ethanol are required.

The difference in concentration dependence for the inhibition of protein synthesis could reflect differences in the precise mechanism by which translation initiation is inhibited; especially for ethanol relative to the other alcohols. However, these differences are analogous to variations observed in the concentration dependent inhibition of growth (Figs. 1 and 2), with the order of alcohol potency being ethanol < *n*-butanol < isoamyl alcohol. On the basis of this, it seems plausible that the inhibition of protein synthesis could be the cause of the growth inhibition observed for cultures treated with these alcohols. However, it is unclear how the different alcohols impact upon protein synthesis and whether the reduced rate of growth observed is important in the morphogenetic switching of colonies.

### 3.4 Inhibition of translation initiation by alcohols in *C*. *albicans* does not rely upon eIF2α kinase activation

A key mechanism by which global translation initiation is controlled in eukaryotes is via the activation of eIF2α kinases (Dever, 2002; Dever et al., 1992; Hinnebusch, 2005). Like *S*. *cerevisiae*, *C*. *albicans* possesses a single eIF2α kinase gene *GCN2*, and the encoded Gcn2p kinase has been shown to mediate a number of responses to cellular stress (Tournu et al., 2005). Previous studies have highlighted the similarities between *C*. *albicans* and *S*. *cerevisiae* in terms of the Gcn2p-dependent regulation of translation initiation (Sundaram and Grant, 2014a; Tournu et al., 2005). In both organisms, either amino acid starvation or oxidative stress causes the activation of Gcn2p leading to increased phosphorylation of eIF2α and a resultant inhibition of translation initiation. Even though in *S*. *cerevisiae* it has been shown that fusel alcohols target translation in a Gcn2p-independent manner (Ashe et al., 2001), it was important in this current study to independently assess the role of Gcn2p in the response to alcohols in *C*. *albicans*.

Therefore, a *C*. *albicans* homozygous *GCN2* deletion mutant (*gcn2Δ*) was used to assess the requirement for the Gcn2p kinase in the various alcohol-dependent effects described above. Consistent with studies on butanol in *S*. *cerevisiae* (Ashe et al., 2001), the inhibition of either growth or translation initiation by butanol, IAA or ethanol in *C*. *albicans* is insensitive to the deletion of the *GCN2* gene (Figs. 1 and 3). In particular, a sensitive comparison of the polysome profiles shows that there is no obvious difference between the response of a *gcn2Δ* mutant and a wild type strain in terms of inhibition of translation by either fusel alcohols or ethanol (Fig. 3A). Furthermore, quantitation of the change in polysome/monosome ratios caused by the alcohols suggests that *GCN2* is not required for the alcohol-dependent polysomal run-off (Fig. 3B). Therefore, it would appear that the Gcn2p kinase plays little or no role in the inhibition of translation initiation by alcohols in *C*. *albicans*. These results also support a conclusion that treatment with alcohols elicits translational inhibition by a different mechanism to amino acid starvation and oxidative stress.

### 3.5 Alcohols do not cause eIF2α phosphorylation, but do induce *GCN4* expression

Given that fusel alcohols inhibit translation initiation in *S*. *cerevisiae* by targeting the guanine nucleotide exchange factor, eIF2B (Ashe et al., 2001), we set out to investigate if the alcohols exert a similar effect in *C*. *albicans*. A key observation that pointed towards eIF2B as a target for butanol in *S*. *cerevisiae* was the demonstration that translation of a *GCN4* reporter mRNA is up-regulated after butanol addition (Ashe et al., 2001). A variety of cellular stresses that inhibit eIF2B to reduce ternary complex and inhibit translation initiation, also induce *GCN4* mRNA translation (Hinnebusch, 2005; Tripathi et al., 2002).

**Fig. 4:**
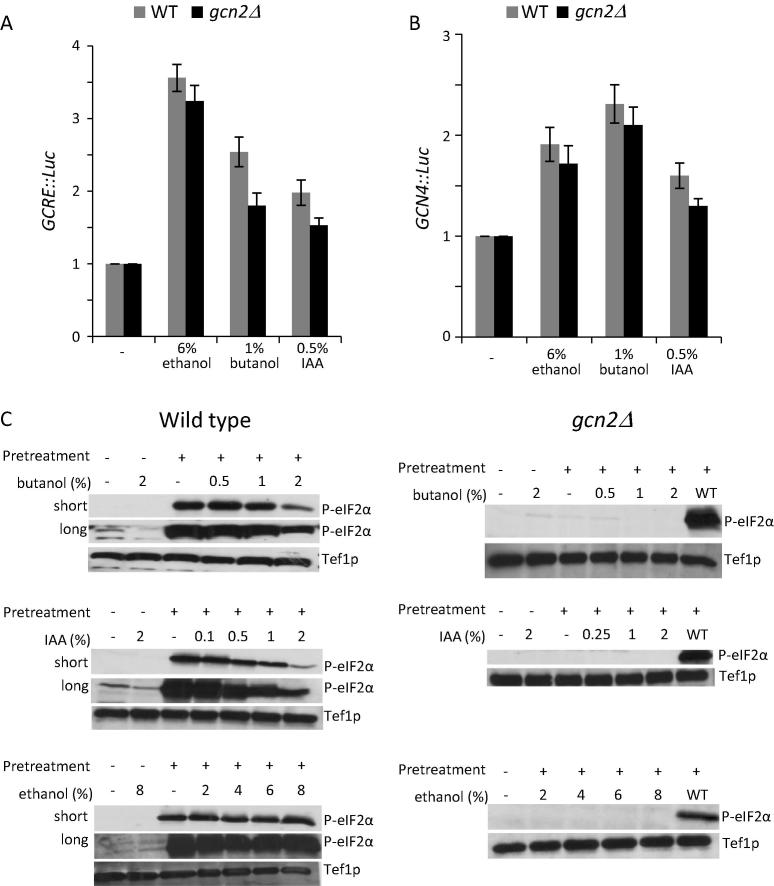
Alcohols do not induce eIF2α phosphorylation, but does induce *GCN4* activation. (A and B) Luciferase assays from strains bearing either *GCRE*:*:Luc* or *GCN4::Luc* reporters treated with the alcohols and concentrations indicated for 2 h. Values are plotted relative to untreated and represent a mean of three biological replicates. Error bars are ±SEM and all the alcohol dependent inductions are statistically significant (*p* < 0.05). (C) Western blots on extracts from wild type and *gcn2Δ* strains probed with antibodies to Tef1p and phospho-eIF2α. Strains were pretreated with 1 mM Cadmium then treated with various concentrations of alcohols (% v/v) for 15 min as indicated. Long and short exposures to film are indicated.

In order to test whether alcohol treatment in *C*. *albicans* induces Gcn4p translation leading to a transcriptional up-regulation of genes harbouring GCRE elements in their promoters, we used strains bearing two different reporter genes. The first reporter contains five copies of the GCRE cloned in a basal promoter upstream of a luciferase reporter gene (Srikantha et al. (1996). Following the addition of 1% butanol, 0.5% IAA or 6% ethanol, there was an increase in *GCRE*-Luc expression relative to untreated controls (Fig. 4A). Similarly, using strains bearing the second reporter, a *GCN4*-Luc reporter construct where the 5′-leader sequence of the *GCN4* gene was cloned upstream of the luciferase gene, all three alcohols used in this study caused induction (Fig. 4B). Variation was seen in the precise level of induction for both reporters depending on the alcohol used, however, a significant induction was always observed (Fig. 4A and B). In *S*. *cerevisiae*, the *GCN4* reporter system is often used as a means to indirectly assess the levels of ternary complex; where the activity of the guanine nucleotide exchange factor eIF2B is a key determinant. As similar observations have been made using the *GCN4* system in *C*. *albicans* (Sundaram and Grant, 2014b), the induction of *GCN4* in response to alcohols is at least consistent with eIF2B being important in this regulation.

Similar to the results for global protein synthesis above, deletion of the *GCN2* gene did not impact significantly upon the level of luciferase induction observed from either reporter for any of the alcohols. Once again, this suggests that neither Gcn2p nor eIF2α phosphorylation is required for the translational effects of butanol, IAA or ethanol in *C*. *albicans*.

However, even though Gcn2p is not required for the inhibition of translation initiation, the induction of Gcn4p prompted us to investigate any changes in the levels of phosphorylated eIF2α following alcohol treatment. Initially, an attempt was made to detect phosphorylated eIF2α in *C*. *albicans* cells using phosphospecific antibodies to eIF2α phosphoserine 51, but a very low basal signal for phosphorylated eIF2α is only just detectable after prolonged exposure for an untreated sample (Fig. 4C, long vs. short). The level of this signal was reduced by butanol treatment, suggesting that eIF2α may have become dephosphorylated. On account of this very low basal level, we decided to investigate the impact of alcohol treatment after a preinduction of eIF2α phosphorylation. In order to achieve a high level of eIF2α phosphorylation prior to alcohol treatment, the strains were first pretreated with 1 mM Cadmium Sulphate which significantly increases eIF2α phosphorylation (Fig. 4C). Subsequent treatment with butanol and IAA cause a dose dependent de-phosphorylation of eIF2α (Fig. 4C). In contrast, no phosphorylation of eIF2α was detected in a gcn2Δ mutant indicating that phosphorylation is entirely dependent on the Gcn2p protein kinase (Fig. 4C). This result is similar to the effects of both fusel alcohols and volatile anaesthetics in *S*. *cerevisiae*, where inhibition of translation initiation also leads to dephosphorylation of eIF2α (Palmer et al., 2005).

Therefore, it appears that in both *C*. *albicans* and *S*. *cerevisiae* the alcohols are activating some phosphatase activity to dephosphorylate eIF2. Indeed previous work in *S*. *cerevisiae* suggests that a type 2A-related phosphatase (Sit4p) might be somehow involved in the dephosphorylation of eIF2α after exposure to fusel alcohols (Taylor et al., 2010). In sharp contrast to the effects of the fusel alcohols, ethanol had little or no observable effect on the level of phospho-eIF2α (Fig. 4C). If, as suggested by the *GCRE*/*GCN4* reporter assays, the alcohols all inhibit translation initiation by a similar mechanism, the fact that they vary in their capacity to induce eIF2α dephosphorylation once again suggests that the dephosphorylation has no bearing on the control of protein synthesis. Therefore, our interpretation of these data is that fusel alcohols induce eIF2α dephosphorylation by activating a phosphatase pathway that is not induced by ethanol, but this pathway is not required for the translational regulation.

### 3.6 eIF2B is the likely translational target of alcohol treatment in *C*. *albicans*

eIF2B is a guanine nucleotide exchange factor that has a critical role in the formation of ternary complex. Previous work has implicated eIF2B as the target for the alcohol regulation of protein synthesis in *S*. *cerevisiae*. A key observation concerns the dynamic localisation of eIF2B in response to alcohols (Taylor et al., 2010). In *S*. *cerevisiae* eIF2B is localised to a single large cytoplasmic body termed the ‘2B body’, where guanine nucleotide exchange has been suggested to occur (Campbell et al., 2005). The 2B body was also found to be dynamic; transiting all areas of the cytoplasm, and alcohol treatment limits this movement (Taylor et al., 2010).

**Fig. 5:**
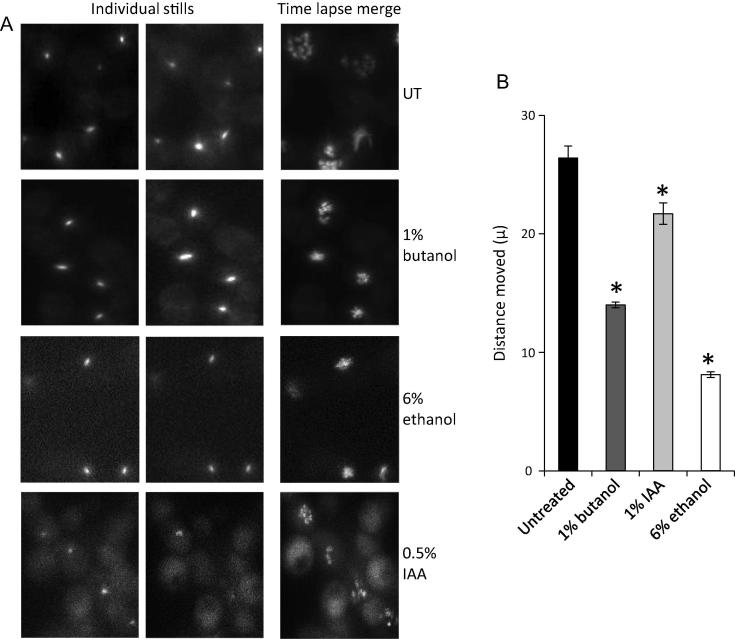
Fusel alcohols and ethanol impede the movement of eIF2B bodies. (A) Images from time-lapse microscopy studies using an eIF2Bγ-GFP expressing *C*. *albicans* strain. The strains were incubated in media with 1% butanol, 0.5% IAA or 6% ethanol, or they were left untreated (UT) for 15 min as indicated. Each row contains two stills from a series of 25 images over a period of 2 min, as well as a merged image of all 25 stills, which serves to depict the total extent of 2B body movement. (B) Bar chart depicting the mean distance moved in *μ* over a 2-min period from 24 time-lapse experiments. Error bars, ±1 SEM; ^∗^ *p* < 0.01.

We generated heterozygous strains of *C*. *albicans* CAI4 strain where one of the genomic copies of the *GCD1* gene (encoding the γ subunit of eIF2B) is C-terminally tagged with GFP. Using epifluorescence microscopy, we observed that, in *C*. *albicans*, eIF2B localises to a large cytoplasmic body in a similar manner to what has been observed in *S*. *cerevisiae* (Fig. 5A). It has been shown in *S*. *cerevisiae* that butanol impedes the movement of the 2B body in a manner that correlates with the sensitivity/resistance of strains (Taylor et al., 2010). To investigate whether butanol, IAA or ethanol impact on the dynamics of the 2B body in *C*. *albicans*, we performed time lapse microscopy. In all, 25 images were collected over a 2-min period and the 2B bodies were tracked to follow their movement over the time course. Quantitation of the total distance moved over the time course reveals that 1% butanol causes a decrease of 50%, while 6% ethanol reduces the movement by about 70% (Fig. 5B). IAA caused a less pronounced yet still significant reduction in the movement of the eIF2B body. The observation that the 2B body moves less after exposure to concentrations of these alcohols, which also inhibit translation initiation, correlates with the possibility that at least part of the inhibition of translation initiation is linked to increased tethering of the 2B body.

### 3.7 Alcohols still regulate protein synthesis and promote filamentation in *Candida* signalling pathway mutants

One of the key virulence factors of *C*. *albicans* is its ability to undergo reversible morphological transitions (Lo et al., 1997; Odds, 1994). Several signalling pathways control morphogenesis in *C*. *albicans*. These include the MAPK pathway which is dependent upon Cph1p, a homologue of *S*. *cerevisiae* Ste12p (Liu et al., 1994), and the Ras-cAMP signalling pathway which requires functional Efg1p (Bockmühl and Ernst, 2001). Both pathways are thought to activate filamentous growth in response to starvation or serum. Transitions from yeast to filamentous growth are therefore compromised in *cph1*/*cph1* or *efg1*/*efg1* mutants (Lo et al., 1997).

**Fig. 6:**
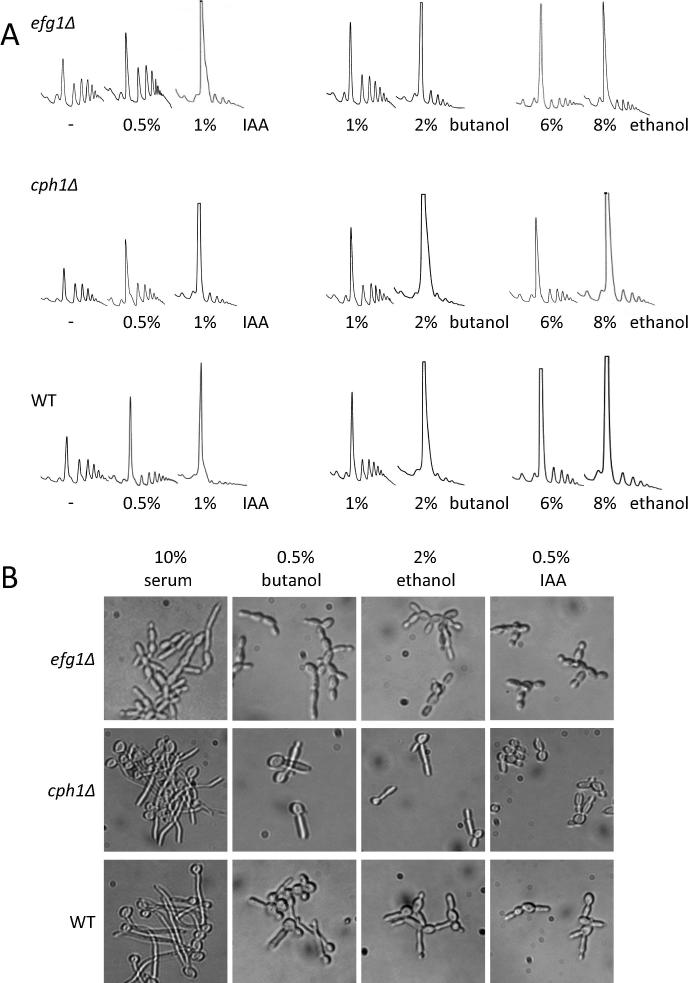
Separate alcohols affect morphological transcriptional regulator mutants. (A) Figure shows polysome analyses assessing the effect of alcohols on translation initiation in *efg1Δ* and *cph1Δ* mutant strains of *C*. *albicans*. Yeast strains were grown in YPD and various concentrations of alcohols were added as indicated for 15 min prior to extract preparation. Extracts were sedimented on 15–50% sucrose gradients and the absorbance at 254 nm was continuously measured. (B) Figure shows images of the mutant strains relative to wild type after treatment in liquid culture for 3 h with various alcohols, as indicated.

In order to assess whether there is any correlation between the activities of these transcription factors and the translational response of *C*. *albicans* to fusel alcohols or ethanol, we analysed the sensitivity of the *efg1Δ* and the *cph1Δ* mutant strains to varying concentrations of the alcohols. In terms of protein synthesis, assessed via polysome analysis, the *cph1Δ* mutant responds to the alcohols in a similar manner to the wild type (Fig. 6A). For the *efg1Δ* mutant, while the response to ethanol is very similar to wild type, this mutant exhibits slight resistance to IAA and butanol (Fig. 6A). Equally, in terms of morphogenesis, especially in the *efg1Δ* mutant, the response to butanol and IAA is marginally less robust early after treatment (Fig. 6B). However, overall it is clear that in the two mutants not only is translation still inhibited by all three alcohols but also the alcohols still cause changes in morphogenesis (Fig. 6B). This result suggests that alcohols might elicit a switch from vegetative to filamentous growth in a pathway that does not rely solely on either of the transcription factors mutated in these strains. Furthermore, given the lack of connection between these pathways, it remains entirely plausible that the regulation of protein synthesis by alcohols could represent a key determinant in the induction of filamentation that primes cells for changes in gene expression at the transcriptional level.

### 3.8 Delicate inhibition of protein synthesis impacts upon morphogenesis

In order to further characterise any link between the regulation of protein synthesis and the switch to filamentous growth, we studied the effects of cycloheximide (CHX) on morphological differentiation. CHX is a widely used inhibitor of eukaryotic protein synthesis that blocks the elongation phase of eukaryotic translation by interfering with deacylated tRNA release from the ribosome to block eEF2-mediated translocation (Schneider-Poetsch et al., 2010). In *S*. *cerevisiae*, CHX inhibits translation across many strains, however resistant mutations are possible that can mean greater concentrations are required for inhibition (Fried and Warner, 1982). Indeed, in the *Candida* or *Kluyveromyces* genera, a polymorphism in the RPL41A gene has been implicated in their CHX resistance (Dehoux et al., 1993; Kawai et al., 1992). As a result high concentrations 1 mg/ml CHX are necessary in *C*. *albicans* to inhibit protein synthesis (data not shown).

**Fig. 7:**
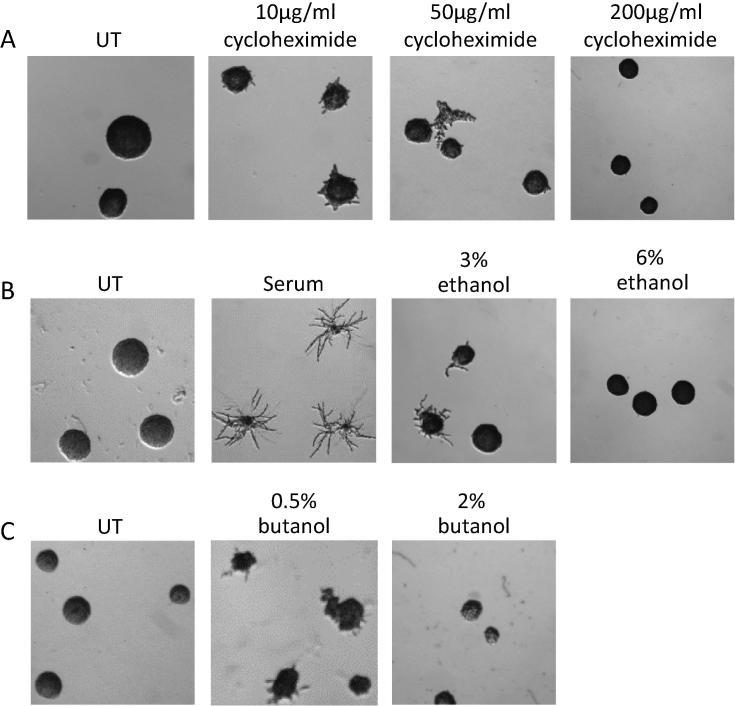
Inhibition of protein synthesis by alcohols also impacts on filamentation in *C. albicans.* Figure shows micro-colonies formed after exponentially grown cells were plated on solid media containing the indicated supplements: (A) cycloheximide, (B) serum or ethanol, and (C) butanol, then grown for 1 day.

In order to study the effects of CHX on morphological differentiation in *C*. *albicans*, we tested various dilutions of CHX. Interestingly, lower sub-lethal concentrations of CHX, 10–100 μg/ml induce some filamentation, but as the concentration increase beyond 200 μg/ml, cells grow in the yeast form as regular colonies (Fig. 7A). All of the concentrations used are insufficient to completely inhibit growth. These observations are similar to the impact of fusel alcohols or ethanol on *C*. *albicans*, where lower concentrations that result in slight alterations to translation, induce pseudohyphal formation, while higher concentrations induce yeast form growth (Fig. 7B and C). On the basis of these results, it appears that low concentrations of these chemicals have a dual effect; they mildly inhibiting protein synthesis and induce morphological differentiation.

## 4 Discussion

Microorganisms are known to produce a range of molecules that regulate their growth, morphology, metabolism and virulence. In the genera *Candida* and *Saccharomyces*, various secreted alcohols have been characterised from culture supernatants, these include: fusel alcohols, aromatic alcohols and ethanol (Martins et al., 2007; Webb and Ingraham, 1963). In this study, we show that *C*. *albicans* produces both ethanol and fusel alcohols (isoamyl alcohol and isobutanol) under both aerobic and semi-anaerobic culture conditions. We demonstrate that such alcohols inhibit growth and protein synthesis at high concentrations via a mechanism that likely results from a targeted regulation of eIF2B. At sub-inhibitory concentrations these alcohols lead to changes in morphogenesis, in that they promote filamentation. Indeed sub-inhibitory concentrations of other agents that target protein synthesis, such as cycloheximide, also lead to the same effect. These results are suggestive that weak regulation of protein synthesis, where the presumed focus is proteome alteration, appears to contribute to the morphogenetic switch from vegetative to filamentous growth in response to alcohols.

The fact that alcohols promote morphogenetic change is not unprecedented. In our study, we show that addition of ethanol (1–3%), butanol (0.5–1%) or isoamyl alcohol (0.1–0.5%) induces pseudohyphal formation in *C*. *albicans* both in liquid and solid media. Previous work has also shown that the addition of fusel alcohols (butanol or isoamyl alcohol) to both *C*. *albicans* and *S*. *cerevisiae* can induce a range of morphological effects, including filament formation (Dickinson, 1996; Lorenz et al., 2000). In terms of the impact of ethanol on *C*. *albicans*, previous studies are somewhat perplexing: ethanol has been described to induce germ tube formation (Pollack and Hashimoto, 1985; Zeuthen et al., 1988) yet at 4%, ethanol inhibits this process (Chauhan et al., 2011; Chiew et al., 1982) Furthermore, concentrations of ethanol ranging from 0.06% to 8% reduce the level of germ tube formation observed in response to a range of inducing conditions (Chauhan et al., 2011). It is entirely possible that many of the differences observed in the response to alcohols could stem from the different culture conditions employed in the various filamentation assays. Our results equate well with the fact that a range of alcohols have been found to impact upon filamentation at physiological concentrations and they show that conditions/concentrations of alcohol that cause a very weak regulation of protein synthesis favour filamentation, whereas more severe regulatory conditions prevent the response.

The fact that alcohols inhibit protein synthesis in *C*. *albicans* is directly comparable to our work in *S*. *cerevisiae* (Ashe et al., 2001). We have shown previously that fusel alcohols (butanol in particular) cause a rapid inhibition of protein synthesis via inhibition of eIF2B and reduced ternary complex levels. As a consequence translation of the *GCN4* mRNA is induced resulting in activation of the general control pathway. Here we show that fusel alcohols cause very similar effects in *C*. *albicans*. Indeed, as in *S*. *cerevisiae*, eIF2B resides in a large cytoplasmic granule in *C*. *albicans* and in response to butanol, the movement of this body is impeded. One of the key differences between this study investigating the response of *C*. *albicans* to alcohols and our previous studies on *S*. *cerevisiae* is the response to ethanol. In *S*. *cerevisiae*, ethanol can inhibit translation initiation but at much higher levels and does not appear to target eIF2B to the same extent, whereas here the response to ethanol is almost identical to that of the two fusel alcohols. This makes sense in terms of the precise metabolic and physiological make-up of the two yeasts. For instance, unlike *S*. *cerevisiae*, *C*. *albicans* is a Crabtree negative yeast (Rozpȩdowska et al., 2011) and hence has not evolved to primarily ferment glucose to ethanol, but instead relies largely upon the aerobic metabolism of glucose. Therefore, it perhaps not surprising that in this yeast the response to ethanol is very similar to the response to other alcohols in that translation is inhibited at relatively low concentrations. In contrast, *S*. *cerevisiae* has evolved mechanisms to maintain translational function even at higher concentrations of ethanol. Taken altogether, these results suggest that generally alcohols could be targeting eIF2B to exert a specific effect on translation initiation in *C*. *albicans*.

A variety of connections between the regulation of protein synthesis and alterations in cell fate such as morphogenesis, differentiation or the response to cellular stress have recently emerged. For instance, low concentrations of cycloheximide alter translation to promote cAMP-dependent morphological differentiation of rat C6 glioma cells (Liu et al., 2010). Equally alterations in ribosome biogenesis impact on Drosophila female germline stem cell differentiation, where reduced rRNA production and presumably reduced protein synthetic capacity induces differentiation (Zhang et al., 2014). Furthermore, studies on the relative contributions of different stages in gene expression during monocyte to macrophage differentiation identify the rate of protein synthesis as a principal regulator (Kristensen et al., 2013). Even in single-celled eukaryotes such as *S*. *cerevisiae* minimal cycloheximide treatment results in a lower proportion of pseudohyphal cells (Kron et al., 1994). In addition, the disruption of established mechanisms of translation repression via the deletion of the genes for either the yeast eIF4E binding proteins (Eap1p or Caf20p) or *GCN2* inhibits the capacity of *S*. *cerevisiae* to undergo filamentous growth in response to nitrogen limitation (Ibrahimo et al., 2006). More recent work in *C*. *albicans* suggests that translational regulation of the *UME6* mRNA specifically may be involved in the morphogenetic switch (Childers et al., 2014).

Therefore, it is entirely plausible that alcohol accumulation represents a stress condition where at the concentrations that accumulate in cells, the impact on global rates of protein synthesis is quite minimal. However, such effects are sufficient to alter the precise selection of specific mRNAs for translation. Such a mechanism would lead to a rapid switch in the cell’s proteome to maximise the timeliness of a cell’s response to specific external cues. More extreme conditions where higher concentrations of alcohol are encountered would alter global protein synthesis rates more dramatically and as such, cells would be targeted for destruction pathways.

## Figures and Tables

**Table 1:** Yeast strains used in this study.

Strain name	Genotype	Source
CAI4	*ura3::λimm434*/*ura3::λimm434*	Fonzi and Irwin (1993)
*gcn2Δ*	*ura3::λimm434*/*ura3::λimm434 gcn2::hisG*/*gcn2::hisG*	Tournu et al. (2005)
yMK2313	*ura3::λimm434*/*ura3::λimm434 GCD1*-*GFP::NAT*/*GCD1*	This study
*efg1Δ*	*ura3::λimm434*/*ura3::λimm434 efg1::hisG*/*efg1::hisG*-*URA3*-*hisG*	Lo et al. (1997)
*cph1Δ*	*ura3::λimm434*/*ura3::λimm434 cph1::hisG*/*cph1::hisG*-*URA3*-*hisG*	Liu et al. (1994)

## References

[b0005] Ashe M.P. (2000). Glucose depletion rapidly inhibits translation initiation in yeast. Mol. Biol. Cell.

[b0010] Ashe M.P. (2001). A novel eIF2B-dependent mechanism of translational control in yeast as a response to fusel alcohols. EMBO J..

[b0015] Biswas S. (2007). Environmental sensing and signal transduction pathways regulating morphopathogenic determinants of Candida albicans. Microbiol. Mol. Biol. Rev..

[b0020] Bockmühl D.P., Ernst J.F. (2001). A potential phosphorylation site for an A-type kinase in the Efg1 regulator protein contributes to hyphal morphogenesis of Candida albicans. Genetics.

[b0025] Brown A.J., Gow N.A. (1999). Regulatory networks controlling Candida albicans morphogenesis. Trends Microbiol..

[b0030] Campbell S.G. (2005). Dynamic cycling of eIF2 through a large eIF2B-containing cytoplasmic body: implications for translation control. J. Cell Biol..

[b0035] Chauhan N.M. (2011). A morphogenetic regulatory role for ethyl alcohol in Candida albicans. Mycoses.

[b0040] Chauhan N.M. (2013). Effect of alcohols on filamentation, growth, viability and biofilm development in Candida albicans. Braz. J. Microbiol..

[b0045] Chen H., Fink G.R. (2006). Feedback control of morphogenesis in fungi by aromatic alcohols. Gene Dev..

[b0050] Chen H. (2004). Tyrosol is a quorum-sensing molecule in Candida albicans. Proc. Natl. Acad. Sci. U.S.A..

[b0055] Chiew Y.Y. (1982). The effects of ergosterol and alcohols on germ-tube formation and chitin synthase in Candida albicans. Can. J. Biochem..

[b0060] Childers D.S. (2014). A 5′ UTR-mediated translational efficiency mechanism inhibits the Candida albicans morphological transition. Mol. Microbiol..

[b0065] Csank C., Haynes K. (2000). Candida glabrata displays pseudohyphal growth. FEMS Microbiol. Lett..

[b0070] Dehoux P. (1993). Natural cycloheximide resistance in yeast. Eur. J. Biochem..

[b0075] Dever T.E. (2002). Gene-specific regulation by general translation factors. Cell.

[b0080] Dever T.E. (1992). Phosphorylation of initiation factor 2 alpha by protein kinase GCN2 mediates gene-specific translational control of GCN4 in yeast. Cell.

[b0085] Dickinson J.R. (1996). ‘Fusel’ alcohols induce hyphal-like extensions and pseudohyphal formation in yeasts. Microbiology.

[b0090] Dickinson J.R. (1998). An investigation of the metabolism of valine to isobutyl alcohol in Saccharomyces cerevisiae. J. Biol. Chem..

[b0095] Dickinson J.R. (2003). The catabolism of amino acids to long chain and complex alcohols in Saccharomyces cerevisiae. J. Biol. Chem..

[b0100] Feng Q. (1999). Ras signaling is required for serum-induced hyphal differentiation in Candida albicans. J. Bacteriol..

[b0105] Fonzi W.A., Irwin M.Y. (1993). Isogenic strain construction and gene mapping in Candida albicans. Genetics.

[b0110] Fried H.M., Warner J.R. (1982). Molecular cloning and analysis of yeast gene for cycloheximide resistance and ribosomal protein L29. Nucleic Acids Res..

[b0115] Gancedo J.M. (2001). Control of pseudohyphae formation in Saccharomyces cerevisiae. FEMS Microbiol. Rev..

[b0120] Ghosh S. (2008). Regulation of aromatic alcohol production in Candida albicans. Appl. Environ. Microbiol..

[b0125] Gimeno C.J. (1992). Unipolar cell divisions in the yeast S. cerevisiae lead to filamentous growth: regulation by starvation and RAS. Cell.

[b0130] Guthrie C., Fink G.R. (1991). Guide to Yeast Genetics and Molecular Biology.

[b0135] Hartwell L.H., McLaughlin C.S. (1969). A mutant of yeast apparently defective in the initiation of protein synthesis. Proc. Natl. Acad. Sci. U.S.A..

[b0140] Hazelwood L.A. (2008). The Ehrlich pathway for fusel alcohol production: a century of research on Saccharomyces cerevisiae metabolism. Appl. Environ. Microbiol..

[b0145] Hershey J.W., Merrick W.C. (2000). Pathway and mechanism of initiation of protein synthesis. Cold Spring Harbor Monogr. Ser..

[b0150] Hinnebusch A.G. (2005). Translational regulation of GCN4 and the general amino acid control of yeast. Annu. Rev. Microbiol..

[b0155] Ibrahimo S. (2006). Regulation of translation initiation by the yeast eIF4E binding proteins is required for the pseudohyphal response. Yeast.

[b0160] Kawai S. (1992). Drastic alteration of cycloheximide sensitivity by substitution of one amino acid in the L41 ribosomal protein of yeasts. J. Bacteriol..

[b0165] Kitagaki H. (2007). Ethanol-induced death in yeast exhibits features of apoptosis mediated by mitochondrial fission pathway. FEBS Lett..

[b0170] Kristensen A.R. (2013). Protein synthesis rate is the predominant regulator of protein expression during differentiation. Mol. Syst. Biol..

[b0175] Kron S.J. (1994). Symmetric cell division in pseudohyphae of the yeast Saccharomyces cerevisiae. Mol. Biol. Cell.

[b0180] Lingappa B.T. (1969). Phenethyl alcohol and tryptophol: autoantibiotics produced by the fungus Candida albicans. Science.

[b0185] Liu H. (2001). Transcriptional control of dimorphism in Candida albicans. Curr. Opin. Microbiol..

[b0190] Liu H. (1994). Suppression of hyphal formation in Candida albicans by mutation of a STE12 homolog. Science.

[b0195] Liu X. (2010). Induction of cell cycle arrest at G1 and S phases and cAMP-dependent differentiation in C6 glioma by low concentration of cycloheximide. BMC Cancer.

[b0200] Lo H.-J. (1997). Nonfilamentous C. albicans mutants are avirulent. Cell.

[b0205] Lorenz M.C. (2000). Characterization of alcohol-induced filamentous growth in Saccharomyces cerevisiae. Mol. Biol. Cell.

[b0210] Martins M. (2007). Morphogenesis control in Candida albicans and Candida dubliniensis through signaling molecules produced by planktonic and biofilm cells. Eukaryot. Cell.

[b0215] Natarajan K. (2001). Transcriptional profiling shows that Gcn4p is a master regulator of gene expression during amino acid starvation in yeast. Mol. Cell. Biol..

[b0220] Odds F.C. (1994). Pathogenesis of Candida infections. J. Am. Acad. Dermatol..

[b0225] Palmer L.K. (2005). Inhibition of translation initiation by volatile anesthetics involves nutrient-sensitive GCN-independent and -dependent processes in yeast. Mol. Biol. Cell.

[b0230] Pavitt G.D. (2005). EIF2B, a mediator of general and gene-specific translational control. Biochem. Soc. Trans..

[b0235] Pollack J.H., Hashimoto T. (1985). Ethanol-induced germ tube formation in Candida albicans. J. Gen. Microbiol..

[b0240] Rozpȩdowska E. (2011). Candida albicans–a pre-whole genome duplication yeast–is predominantly aerobic and a poor ethanol producer. FEMS Yeast Res..

[b0245] Schneider-Poetsch T. (2010). Inhibition of eukaryotic translation elongation by cycloheximide and lactimidomycin. Nat. Chem. Biol..

[b0250] Srikantha T. (1996). The sea pansy Renilla reniformis luciferase serves as a sensitive bioluminescent reporter for differential gene expression in Candida albicans. J. Bacteriol..

[b0260] Sudbery P.E. (2011). Growth of Candida albicans hyphae. Nat. Rev. Microbiol..

[b0255] Sudbery P. (2004). The distinct morphogenic states of Candida albicans. Trends Microbiol..

[b0265] Sundaram A., Grant C.M. (2014). Oxidant-specific regulation of protein synthesis in Candida albicans. Fungal Genet. Biol..

[b0270] Sundaram A., Grant C.M. (2014). A single inhibitory upstream open reading frame (uORF) is sufficient to regulate Candida albicans GCN4 translation in response to amino acid starvation conditions. RNA.

[b0275] Taylor E.J. (2010). Fusel alcohols regulate translation initiation by inhibiting eIF2B to reduce ternary complex in a mechanism that may involve altering the integrity and dynamics of the eIF2B body. Mol. Biol. Cell.

[b0280] Tournu H. (2005). Global role of the protein kinase Gcn2 in the human pathogen Candida albicans. Eukaryot. Cell.

[b0285] Tripathi G. (2002). Gcn4 co-ordinates morphogenetic and metabolic responses to amino acid starvation in Candida albicans. EMBO J..

[b0290] Webb A.D., Ingraham J.L. (1963). Fusel oil. Adv. Appl. Microbiol..

[b0295] Zeuthen M.L. (1988). Ethanol tolerance and the induction of stress proteins by ethanol in Candida albicans. J. Gen. Microbiol..

[b0300] Zhang Q. (2014). Changes in rRNA transcription influence proliferation and cell fate within a stem cell lineage. Science.

